# Complex Response of the Chlorarachniophyte Bigelowiella natans to Iron Availability

**DOI:** 10.1128/mSystems.00738-20

**Published:** 2021-02-09

**Authors:** Eva Kotabova, Ronald Malych, Juan José Pierella Karlusich, Elena Kazamia, Meri Eichner, Jan Mach, Emmanuel Lesuisse, Chris Bowler, Ondřej Prášil, Robert Sutak

**Affiliations:** a Institute of Microbiology, Academy of Sciences, Centrum Algatech, Trebon, Czech Republic; b Department of Parasitology, Faculty of Science, Charles University, BIOCEV, Vestec, Czech Republic; c Institut de Biologie de l'ENS, Département de Biologie, École Normale Supérieure, CNRS, INSERM, Université PSL, Paris, France; d Jacques Monod Institute, UMR7592 CNRS, Paris Diderot University, Paris, France; e CNRS Research Federation for the Study of Global Ocean Systems Ecology and Evolution, FR2022/Tara Oceans GOSEE, Paris, France; Scripps Institution of Oceanography

**Keywords:** *Bigelowiella natans*, iron, metagenomics, metatranscriptomics, photosynthesis, phytoplankton, proteomics

## Abstract

The productivity of the ocean is largely dependent on iron availability, and marine phytoplankton have evolved sophisticated mechanisms to cope with chronically low iron levels in vast regions of the open ocean. By analyzing the metabarcoding data generated from the *Tara* Oceans expedition, we determined how the global distribution of the model marine chlorarachniophyte Bigelowiella natans varies across regions with different iron concentrations. We performed a comprehensive proteomics analysis of the molecular mechanisms underpinning the adaptation of *B. natans* to iron scarcity and report on the temporal response of cells to iron enrichment. Our results highlight the role of phytotransferrin in iron homeostasis and indicate the involvement of CREG1 protein in the response to iron availability. Analysis of the *Tara* Oceans metagenomes and metatranscriptomes also points to a similar role for CREG1, which is found to be widely distributed among marine plankton but to show a strong bias in gene and transcript abundance toward iron-deficient regions. Our analyses allowed us to define a new subfamily of the CobW domain-containing COG0523 putative metal chaperones which are involved in iron metabolism and are restricted to only a few phytoplankton lineages in addition to *B. natans*. At the physiological level, we elucidated the mechanisms allowing a fast recovery of PSII photochemistry after resupply of iron. Collectively, our study demonstrates that *B. natans* is well adapted to dynamically respond to a changing iron environment and suggests that CREG1 and COG0523 are important components of iron homeostasis in *B. natans* and other phytoplankton.

**IMPORTANCE** Despite low iron availability in the ocean, marine phytoplankton require considerable amounts of iron for their growth and proliferation. While there is a constantly growing knowledge of iron uptake and its role in the cellular processes of the most abundant marine photosynthetic groups, there are still largely overlooked branches of the eukaryotic tree of life, such as the chlorarachniophytes. In the present work, we focused on the model chlorarachniophyte *Bigelowiella natans*, integrating physiological and proteomic analyses in culture conditions with the mining of omics data generated by the *Tara* Oceans expedition. We provide unique insight into the complex responses of *B. natans* to iron availability, including novel links to iron metabolism conserved in other phytoplankton lineages.

## Abstract (precis)

Despite low iron availability in the ocean, marine phytoplankton require considerable amounts of iron for their growth and proliferation. While there is a constantly growing knowledge of iron uptake and its role in the cellular processes of the most abundant marine photosynthetic groups, there are still largely overlooked branches of the eukaryotic tree of life, such as the chlorarachniophytes.

## INTRODUCTION

Iron is undoubtedly one of the key nutrients influencing the growth of marine phytoplankton, limiting primary production in vast regions of the ocean (reviewed in reference 1). Photoautotrophs are particularly dependent on iron because of the iron-demanding components of the photosynthetic apparatus. Iron starvation results in growth reduction due to its negative effects on several processes, most importantly photosynthesis, the tricarboxylic acid (TCA) cycle, and nitrate assimilation (2, 3).

There are many common and general responses of photosynthesis to iron stress among phytoplankton. Cells reduce the concentration of light-harvesting pigments but often synthesize unusual light-harvesting complexes that seem to compensate for the loss of iron-rich reaction centers or, in a decoupled form, serve as temporary storage of pigments during periods of iron stress. Iron deficiency induces remodeling of photosynthetic membranes to match the iron requirements of individual photosynthetic components (reviewed in reference 3). Photosystem I (PSI) is in general preferentially downregulated relative to PSII and cytochrome *b*_6_*f* (3, 4). Iron stress also results in downregulation of carbon fixation capacity and respiration, although to a lesser extent than thylakoid membrane processes (5).

Even though our knowledge of the adaptation to low iron availability in the ocean has improved significantly in recent years, we are still far from understanding the molecular principles behind these mechanisms. There are some notable exceptions. The first is an extraordinarily efficient iron uptake system recently described in diatoms involving phytotransferrin (6, 7). The second is the use of ferritin proteins for iron storage during periods of iron supplementation (8, 9). Last is the replacement of iron-dependent enzymes by their iron-free isofunctional counterparts, for example, the photosynthetic electron shuttles: the replacement of iron-sulfur cluster-containing ferredoxin by flavodoxin or of cytochrome *c*_6_ by the cuproprotein plastocyanin (10–13). Further investigation and introduction of new marine model species to study iron metabolism are of great importance considering the changes in ocean iron cycles expected due to ongoing ocean acidification and warming (14) and controversially discussed proposals to use ocean iron fertilization as a means to stimulate carbon dioxide sequestration from the atmosphere (15).

One such group of largely overlooked unicellular marine microalgae are the chlorarachniophytes, which belong to the Rhizaria, one of the most diverse and abundant, yet least studied, groups of eukaryotes (16). Chlorarachniophytes represent one of the two relatively small subgroups of photosynthetic rhizarians (16). They have been collected from diverse environments, including sandy beaches as well as ocean surface seawaters. Their life cycles vary and can comprise one or more cell forms—amoeboid, coccoid, and flagellate. To date, the research interest in chlorarachniophytes has been largely in the study of their unique plastids, which harbor a vestigial nucleus that is absent in most cases of secondary endosymbiosis (17).

The model chlorarachniophyte Bigelowiella natans is a small mixotrophic flagellate, sometimes producing ameboid pseudopodia. Its secondary plastid is of green-algal origin (18). Little is known about the physiology of *B. natans*, but complete annotation of its genome (19) allowed pioneering studies, which so far have focused on the function of its photosynthetic apparatus, its diurnal transcriptional regulation, and its response to light stress (20–22). A close analysis of its genome can also reveal genetic adaptations to iron availability fluctuations. *B. natans* contains both terminal acceptors of the photosynthetic electron transport chain: ferredoxin and flavodoxin (13). In addition, it has the three superoxide dismutase families (NiSOD, CuSOD, and Fe/MnSOD) (23). However, it exclusively relies on cytochrome *c*_6_ to transport electrons toward photosystem I, as it lacks any gene for plastocyanin (23). Finally, it lacks the iron storage protein ferritin.

In this study, we aimed to elucidate the effect of iron availability on *B. natans*. We chose this species because of its emerging role as a model microalga, its easy cultivation, and its clear physiological response to iron limitation under laboratory conditions. Using proteomic, biochemical, and physiological techniques, we revealed how iron availability affects photosynthesis in *B. natans*, shedding light on the mechanisms cells employ to cope with iron deprivation and how they respond to subsequent iron repletion. By analyzing the molecular data generated by the *Tara* Oceans expedition (24, 25), we elucidated the global distribution of *B. natans* across ocean regions of different iron levels. Additionally, we used the *Tara* Oceans metagenomes and metatranscriptomes (26) to assess the ecological prevalence of one key molecular player, the gene *CREG1*, in microalgal communities, which we identified as an important component of the *B. natans* iron response in our laboratory studies. Finally, we identified a potential new subfamily of metallochaperones involved in iron metabolisms in specific phytoplankton lineages.

## RESULTS

**FIG 1:**
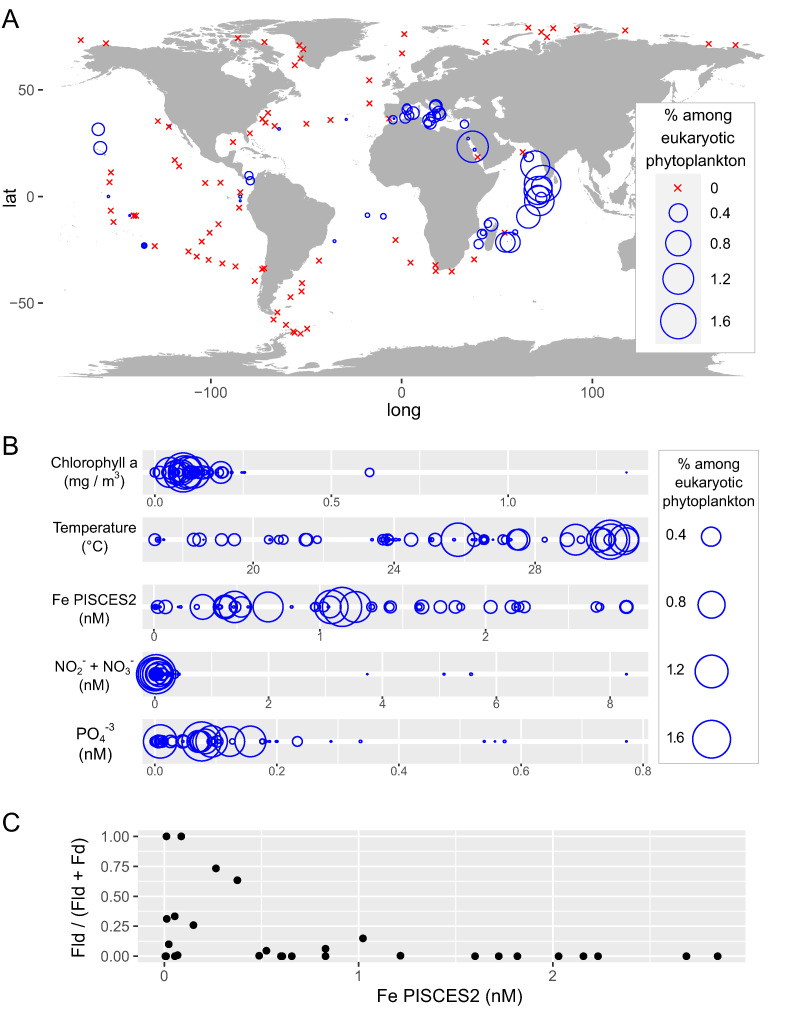
Global biogeographical and environmental distribution of *Bigelowiella natans* in surface samples across *Tara* Oceans stations. (A) *B. natans* biogeography based on 18S rRNA gene (V9 region) metabarcoding data (24, 25). The bubble size represents barcode abundance, while crosses indicate the absence of sequences at a particular sampling site. Abundances are given as percentages of the total phytoplankton barcodes (>85% identity to phytoplankton sequences in reference databases, which corresponds to 21% total eukaryotic barcodes in the analyzed samples). (B) *B. natans* abundance distribution according to environmental parameters. Bubble sizes are displayed as in the previous panel. (C) Flavodoxin (Fld)/ferredoxin (Fd) transcript ratios for the *Bigelowiella* genus in *Tara* Oceans metatranscriptomes according to predicted environmental concentrations of iron. The analyzed metabarcoding and metatranscriptomic data sets correspond to samples from the size fractions 0.8 to 2,000 μm and 0.8 to 5 μm, where *B. natans* is most abundant.

We carried out the first global ocean biogeographical and environmental distribution analysis of *B. natans* using the *Tara* Oceans metabarcoding of the 18S (V9 region) rRNA gene (Fig. 1). *B. natans* reaches up to 1.6% of eukaryotic phytoplankton reads and is detected in locations with temperate waters (>17°C, but especially abundant in >25°C), low macronutrient concentrations (<0.5 nM NO_2_^−^/NO_3_^−^ and <0.2 nM PO_4_^3−^), and low chlorophyll levels (<0.25 mg/m^3^) and thus low algal biomass (Fig. 1B). In relation to iron availability, *B. natans* is distributed in a wide range of concentrations, covering many orders of magnitude of modeled values (Fig. 1). This was also observed when its biogeography was analyzed: we found high abundance in the iron-rich Mediterranean Sea and Red Sea but also in intermediate-iron regions of the Indian Ocean and in low-iron locations of the North Pacific (Fig. 1). Among the potential mechanisms that enable this species to live under such different iron regimens, we analyzed the classic flavodoxin/ferredoxin substitution by carrying out a sequence similarity search in the *Tara* Oceans metatranscriptomes (26). We found a clear increase of the *Bigelowiella* flavodoxin-to-ferredoxin transcript ratio under low iron conditions, showing a strong acclimation response to the metal deficiency (Fig. 1C). Ferredoxin metatranscriptomic reads were detected in almost all geographical sites with the exception of two locations with modeled iron concentrations of less than 0.1 nM, where only flavodoxin was detected. Flavodoxin metatranscriptomic reads are generally detected when modeled iron concentrations are <1 nM, and they are the major component in a few cases when concentrations are <0.4 nM.

To complement the observed iron-driven environmental patterns, we aimed to describe the physiological responses of *B. natans* to iron enrichment under culture conditions. Proteomic analysis is one of the most powerful tools to study the complex cellular responses to changing environmental conditions. In order to identify proteins involved in maintaining iron homeostasis in *B. natans* cells and to observe the dynamics of the response to iron supply, we performed a time course label-free comparative analysis of iron-induced changes in *B. natans* whole-cell proteomes. Iron-limited *B. natans* cultures were supplemented with iron and compared at different time points with cells kept under iron-limited conditions. After 24 h, proteomic profiles were compared with cells grown under long-term iron-sufficient conditions. The data are summarized in Data Set S1; selected proteins discussed here are described in Table 1. We identified 74 proteins whose expression was more than 2-fold higher in iron-sufficient cells than in iron-limited cells; of these, 38 proteins showed >1.5-fold increases in abundance 24 h after addition of iron to iron-limited cells. A total of 84 proteins were downregulated >2-fold in iron-rich cells, and the abundance of 39 of them decreased >1.5-fold within 24 h after iron supply.

**TABLE 1:**
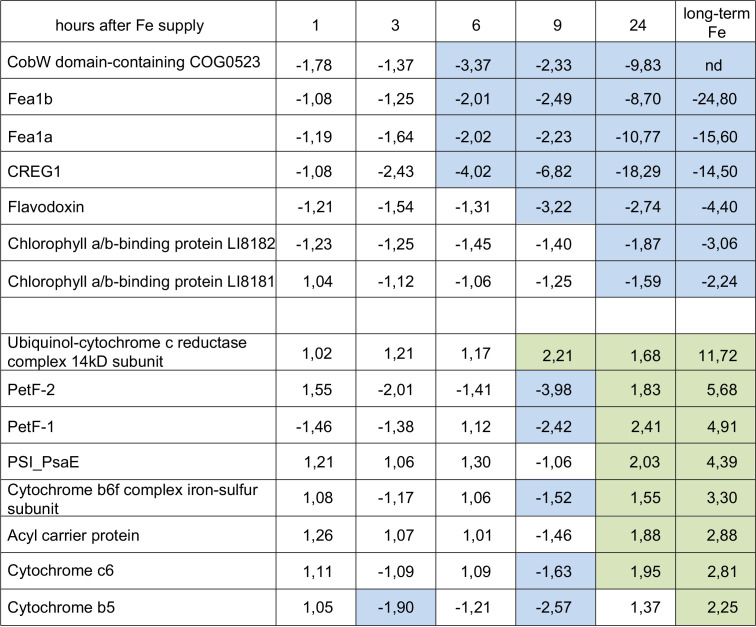
Iron-induced changes in the abundance of selected proteins^*a*^

a Data are fold change in protein abundance in iron-limited cells 1, 3, 6, 9, and 24 h after iron addition. The last column shows changes between iron-limited cells and cells grown under long-term iron-rich conditions. Green color indicates >1.5-fold upregulation compared to iron-limited cells; blue color indicates >1.5-fold downregulation.

10.1128/mSystems.00738-20.1 DATA SET S1 Proteomic analysis of iron-induced changes of *B. natans*. Proteins whose expression changed more than twofold in iron-sufficient cells compared to iron-limited cells are listed. Yellow color indicates proteins downregulated/upregulated more than 1.5-fold 24 h after addition of iron to iron-limited cells. 0, iron-limited cells; −Fe, iron-limited cells after iron enrichment; +Fe, cells grown under long-term iron-rich conditions. Download Data Set S1, XLSX file, 13.6 MB. Copyright © 2021 Kotabova et al. 2021 Kotabova et al. This content is distributed under the terms of the Creative Commons Attribution 4.0 International license.

**FIG 2:**
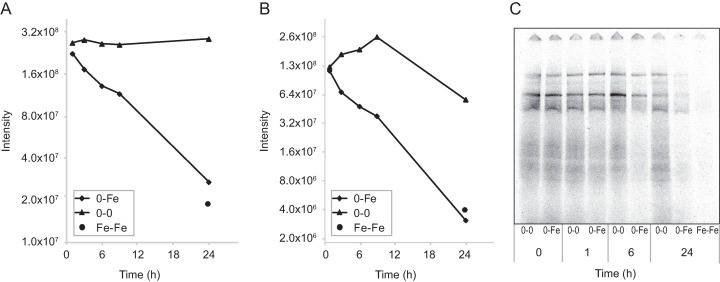
Effect of iron enrichment on *B. natans* iron acquisition machinery and CREG1 abundance. (A and B) Changes in protein levels of *B. natans* phytotransferrin homologue Fea1b (jgi|Bigna1|133614|aug1.22_g8322) (A) and CREG1 protein (jgi|Bigna1|54718|estExt_Genewise1Plus.C_410023) (B) as revealed by comparative whole-cell proteomic analysis. (C) Changes in the incorporation of ^55^Fe into *B. natans* protein complexes determined by blue native electrophoresis separation of total cell extracts. At each time point, extracellular iron was removed, and cells were incubated for 1 h with 1 μM ^55^Fe-citrate. 0-0, iron-limited cells; 0-Fe, iron-limited cells after iron enrichment; Fe-Fe: cells grown under long-term iron-rich conditions.

Not surprisingly, among the proteins most dramatically regulated by iron were two homologues of the Fea1-domain-containing proteins functioning as phytotransferrins, experimentally verified to be responsible for iron utilization in the diatom Phaeodactylum tricornutum (iron starvation-induced protein Isip2a) (6, 7) and the picoalga Ostreococcus tauri (Ot-FEA1) (27, 28). The expression of both proteins dropped 2-fold 6 h after iron supplementation, and the changes reached 8.7-fold and 10.8-fold, respectively, after 24 h. A similar decrease in abundance was observed for a member of the COG0523 family of putative metal chaperones (29, 30) with known functions in binding and trafficking of metals (Zn, Co, and Fe) to various cellular processes. These proteins have a CobW domain and are part of a large multigene family in *B. natans* and in other organisms, but only one member was found to be iron responsive in our analysis (Joint Genome Institute PhycoCosm database ID jgi|Bigna1|43984|e_gw1.87.5.1). A response to iron enrichment of similar magnitude was also observed for CREG1, a protein of unclear function shown to play a role in proliferation and differentiation in multicellular organisms (31). Its abundance 24 h after iron resupply decreased to levels similar to those in long-term iron-replete cells, being more than 10-fold higher under iron-limited conditions (Table 1; Fig. 2B).

Considering the strong iron-dependent regulation of *B. natans* phytotransferrins, we investigated how iron enrichment affects the rate of iron acquisition in time. To achieve this goal, first we removed extracellular iron from the cells at different times after iron addition and then studied how iron radioisotope (^55^Fe) was incorporated into protein complexes following supplementation (Fig. 2). Consistent with the rapid decline in the expression of the phytotransferrins, we found that the iron uptake machinery was significantly attenuated 6 h after iron resupply to iron-limited cells. At 24 h after iron enrichment, only weak incorporation of iron radionuclide to cell proteins was observed, a result similar to that obtained with cells grown under long-term iron-replete conditions.

**FIG 3:**
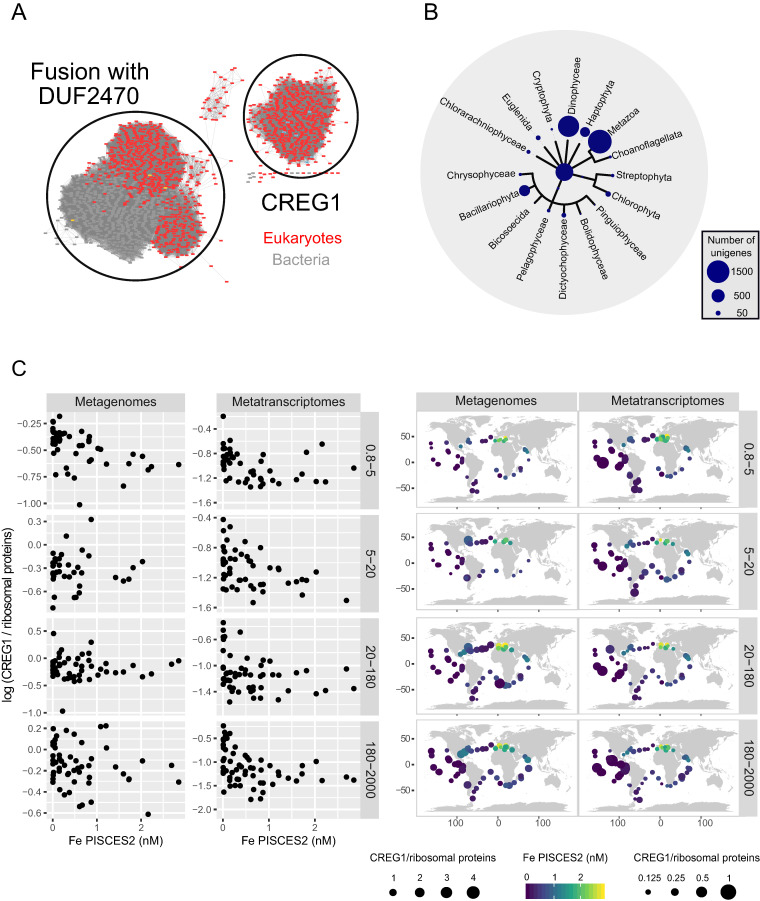
Taxonomic and environmental distribution of *Tara* Oceans unigenes encoding CREG1 protein. (A) Protein similarity network for the Pfam domain PF13883. Two separated clusters were found, one for CREG1 and another for sequences with an extra domain, including pyridoxamine 5′-phosphate oxidases and bacterial HugZ. The network was built with sequences retrieved from the literature and from reference genomes and transcriptomes, and it was used for the selection of *Tara* Oceans unigenes coding for CREG1. (B) Taxonomic distribution of the 4,855 sequences coding for CREG1 in the eukaryotic *Tara* Oceans gene catalogue. The main eukaryotic branches are represented, with the bubble sizes scaled to the number of sequences. Sequences not assigned below the Eukarya domain are shown at the base of the tree. (C) Iron correlations of gene and transcript abundance of *CREG1* across different size-fractionated *Tara* Oceans samples. The read abundances from metagenomes and metatranscriptomes are compared with the modeled iron levels (from PISCES2) obtained from each sampling station (represented by each point). The plankton were separated into discrete size fractions using a serial filtration system. While the size range between 0.8 and 5 μm is enriched in *B. natans* and other small phytoplankton, the size range between 180 and 2,000 μm largely contains chain-forming diatoms and metazoans. Maps on the right show the biogeographical patterns, with bubble size according to read abundance and color according to iron concentrations (scale bar on the right). CREG1 abundance was normalized to the average abundance of 21 conserved single-copy ribosomal proteins from eukaryotes (26).

Taking into account that CREG1 showed one of the strongest responses and that, unlike phytotransferrin, little is known about its role in iron starvation, we decided to focus on it. Thus, we investigated whether the response observed in *B. natans* is also present in natural populations of photosynthetic eukaryotes, by searching for sequences encoding CREG1 in the eukaryotic *Tara* Oceans gene catalogue (26). This catalogue is derived from metagenomes and metatranscriptomes collected during the *Tara* Oceans global circumnavigation from 68 geographical locations across all the major oceanic provinces except the Arctic. We built a protein sequence similarity network for sequences having the same functional domain (Pfam PF13883) as CREG1, which were retrieved from the literature and from reference genomes and transcriptomes (Fig. 3A; also, see Materials and Methods). Based on this network, we were able to recognize the *Tara* Oceans unigenes coding for CREG1. We found a total of 4,855 sequences, ∼33% of them assigned to metazoans and 47% to the main groups of marine photosynthetic eukaryotes, while 19% were not assigned below the domain *Eukarya* (Fig. 3B). The diversity of *CREG1* sequences among phototrophs reflects the abundance of the corresponding taxa; i.e., the highest number of sequences are assigned to dinoflagellates (Dinophyceae), diatoms (Bacillariophyta), and haptophytes (Haptophyta), the three most prolific groups of marine eukaryotic microalgae. A fraction of the signal also emerged from the chlorarachniophytes, corresponding to 41 *CREG1* sequences.

We analyzed the abundances of metagenomic and metatranscriptomic reads mapping to all *CREG1* sequences to provide the gene and transcript levels in each sample. Although we found that *CREG1* is widespread in the ocean, the gene and transcript abundances are strongly anticorrelated with estimated environmental iron concentrations (Fig. 3C). The lowest abundances are observed in the Mediterranean and Arabian Seas, both iron replete due to desert dust deposition, whereas the highest levels are in the tropical Pacific and Southern Oceans, well-known iron-limited regions (Fig. 3C). These tendencies are similar to those observed in the *Tara* Oceans data set with iron marker genes such as iron starvation-induced proteins (ISIPs), although the latter were stronger (32, 33).

**FIG 4:**
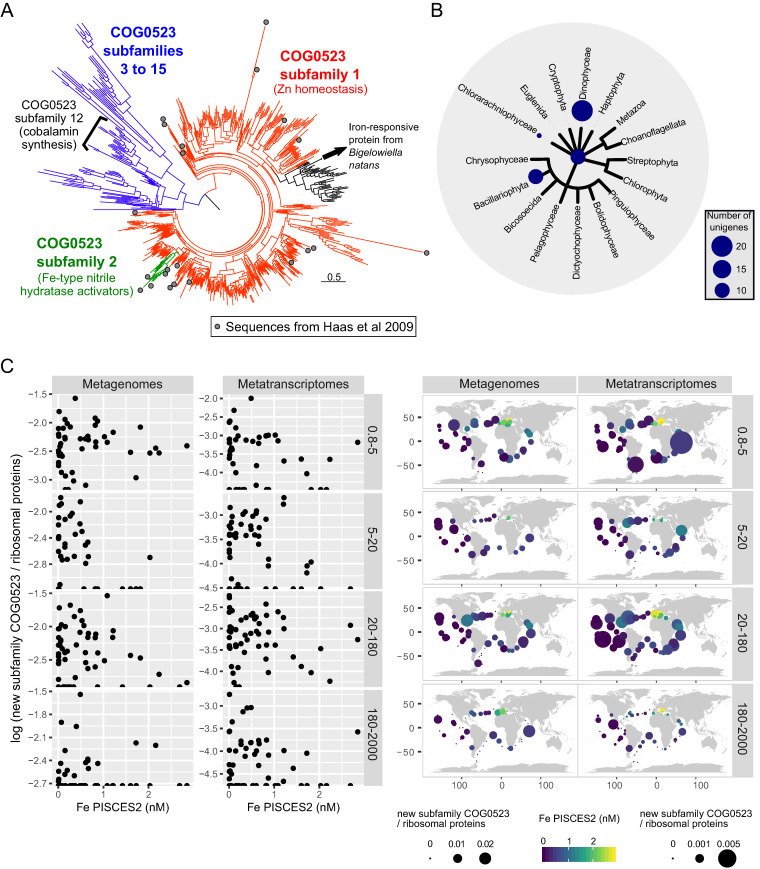
Taxonomic and environmental distribution of *Tara* Oceans unigenes encoding the proposed new COG0523 subfamily. (A) Phylogeny of the Pfam domain CobW (PF02492) of the COG0523 family. The tree was built with sequences retrieved from reference genomes and transcriptomes and from the work of Haas et al. (29). The functions of characteristic subfamilies are indicated. The proposed new COG0523 subfamily corresponds to the black branch. (B) Taxonomic distribution of the 39 sequences encoding the proposed new COG0523 subfamily in the eukaryotic *Tara* Oceans gene catalogue. The main eukaryotic branches are represented, with the bubble sizes corresponding to the number of sequences. Sequences not assigned below the domain *Eukarya* are shown at the base of the tree. (C) Iron correlation analysis of gene and transcript abundance for the proposed new COG0523 subfamily across different size-fractionated *Tara* Oceans samples. The read abundances from metagenomes and metatranscriptomes are compared with the modeled iron levels (PISCES2) at each sampling station (represented by each point). The plankton were separated into discrete size fractions using a serial filtration system. While the size range between 0.8 and 5 μm is enriched in *B. natans* and other small phytoplankton, the size range between 180 and 2,000 μm largely contains chain-forming diatoms and metazoans. Scatterplots display the correlations (left), whereas the maps show the biogeographical patterns (right), with bubble sizes corresponding to read abundance and color to iron concentrations. The abundance of the new COG0523 subfamily was normalized to the average abundance of 21 conserved single-copy ribosomal proteins from eukaryotes (26).

As mentioned above, one of the strongest responses to iron enrichment was observed for the CobW domain-containing protein of the COG0523 family. We made a deeper analysis of the COG0523 family, taking into account that this multigene family of metal chaperones has not been linked previously with iron metabolism. The only exception corresponds to the members of subfamily 2, one of the 15 subfamilies defined by Haas et al. (29), which are involved in the activation of Fe-type nitrile hydratases. In order to determine the subfamily affiliation of the iron-regulated protein 43984 of *B. natans*, we built a comprehensive phylogeny for the CobW domain of the COG0523 family using sequences retrieved from the literature and from reference genomes and transcriptomes (Fig. 4A). Our sequence of interest forms a highly supported clade (aLRT value of 1) with sequences from 7 species of chlorarachniophytes (including *B. natans*), 5 diatoms, 10 dinoflagellates, and 1 euglenid (black branch in Fig. 4A). This branch groups among paraphyletic subfamily 1 (similar to the case of the subfamily 2), and it is sister to a branch of subfamily 1 which includes protein 123019 of Chlamydomonas reinhardtii, whose gene expression increases under zinc-limiting conditions but is unaffected by iron nutrition (29). Therefore, we postulate that protein 43984 of *B. natans* is part of a new COG0523 subfamily that is responsive to low iron in some algal lineages. We further investigated this new subfamily in the eukaryotic *Tara* Oceans gene catalogue (26). Although we found a total of 22,591 sequences coding for CobW domains, only 39 corresponded to this iron-responsive subfamily: 19 from diatoms, 11 from dinoflagellates, 9 from unassigned or unknown eukaryotes, and 1 from chlorarachniophytes (with 99% identity to protein 43984 of *B. natans*) (Fig. 4B), suggesting that its presence is restricted to a few specific lineages among these groups. This is also reflected by a low number of metagenomic and metatranscriptomic reads, but still the highest read abundances are detected in samples with estimated iron concentrations <1 nM (Fig. 4C).

Known indicators of iron nutritional status in marine phytoplankton, ferredoxin and flavodoxin, displayed opposite responses to iron concentrations in *B. natans*; while the expression of flavodoxin decreased, the two ferredoxins were induced in iron-rich cells (Table 1), in agreement with the transcript abundance pattern observed in the environmental populations (Fig. 1C). However, these changes were much less dramatic than was the case for the phytotransferrins COG0523 and CREG1 (Table 1). Several other iron-containing proteins or subunits of iron-dependent complexes, including components of the photosynthetic and respiratory machinery, were significantly upregulated 24 h after iron supply, e.g., cytochrome *c*_6_, cytochrome *b*_5_, and components of cytochrome *bc*_1_ and cytochrome *b*_6_*f* complexes (Table 1; Data Set S1). Interestingly, the induction of iron-dependent proteins was rather slow; the levels were increased in most cases only 24 h after iron enrichment. We observed a similar iron-induced decline in two stress-inducible light-harvesting proteins from the LI818 family of 5 related genes (21, 22) (Table 1). These proteins, also known as LHCX, are known to play a direct role in energy dissipation under stress in secondary endosymbionts of the red lineage. Together these results indicate that the photosynthetic apparatus in iron-deprived *B. natans* cells was not significantly compromised during iron starvation.

**FIG 5:**
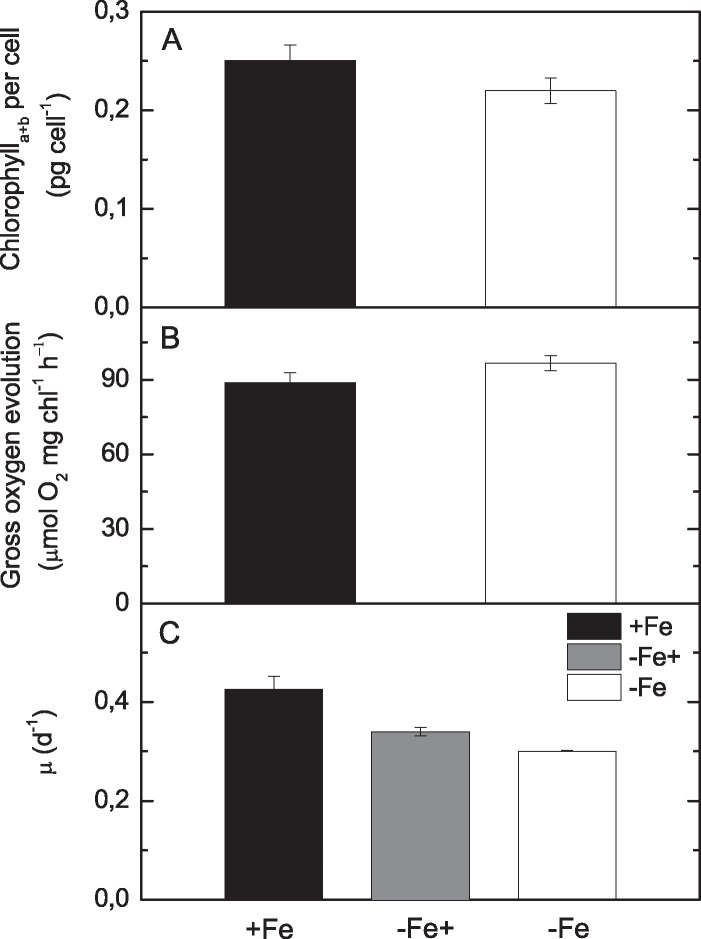
General physiological responses of *B. natans* to iron availability. (A) Chlorophyll (*a+b*) concentrations per cell, (B) oxygen evolution rates, and (C) specific growth rates (μ) of *B. natans* grown in the presence of iron (+Fe), in the absence of iron (−Fe), and in the 7 days following iron supply to previously iron-limited cells (−Fe+). Data are averages and standard deviations (SD); *n* = 3.

**FIG 6:**
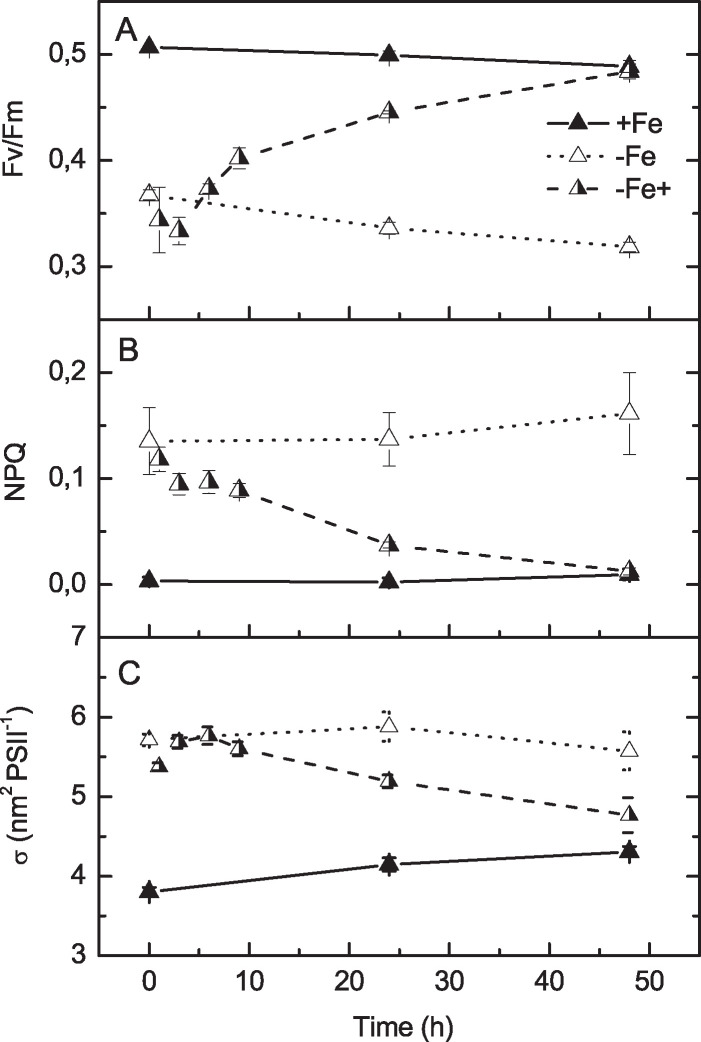
Physiological responses of *B. natans* to iron availability measured using fluorescence techniques. (A) Maximum quantum yield of PSII (*F_v_*/*F_m_*), (B) nonphotochemical fluorescence quenching (NPQ), and (C) effective PSII antenna cross sections (σ_PSII_) of *B. natans* grown in the presence of iron (+Fe), in the absence of iron (−Fe), and after iron supply to iron-limited cells (−Fe+) during the 48 h after iron supply. Data are averages and SD; *n* = 3.

To test for the effects on photosynthesis directly, we complemented our proteomics study with physiology experiments. From a physiological point of view, *B. natans* appears to be well adapted to respond dynamically to changing iron availability and to recover quickly from limitation. We did not observe severe chlorosis of cells, a typical symptom of iron limitation in photoautotrophs (reviewed in reference 3). Chlorophyll concentration per cell was only 13.5% higher in iron-sufficient cultures (Fig. 5A). Cell size was almost the same (3.5% larger size of iron-sufficient cells). Iron-sufficient cultures grew 42% faster than iron-limited ones, but iron enrichment accelerated growth rate by only 13% in the following 7 days (Fig. 5C). Interestingly, the rate of photosynthesis based on oxygen evolution remained unaffected by iron supply (Fig. 5B), while the iron enrichment initiated a substantial increase of photosystem II (PSII) photochemical efficiency (Fig. 6A). The maximum quantum yield of PSII (*F_v_*/*F_m_*) of iron-limited cells increased from ∼0.35 to 0.45 during 24 h after iron enrichment and reached the levels of long-term iron-rich conditions ∼0.50 within 48 h. Iron-limited *B. natans* responded to actinic light (200 μmol photons m^−2^ s^−1^) by dissipation of the excess absorbed energy to heat by increased nonphotochemical fluorescence quenching (NPQ). NPQ then dropped rapidly after iron enrichment, with the same kinetics as increase in *F_v_*/*F_m_* (Fig. 6B). The effect was significant already within the first 3 h, and 48 h later, NPQ was no longer detectable. Additionally, the effective PSII antenna cross-section (σ_PSII_) increased (by 1/3) under iron-limiting conditions (Fig. 6C). The functional PSII antenna size responded to the iron enrichment distinctly slower than the other fluorescence parameters (Fig. 6C).

**FIG 7:**
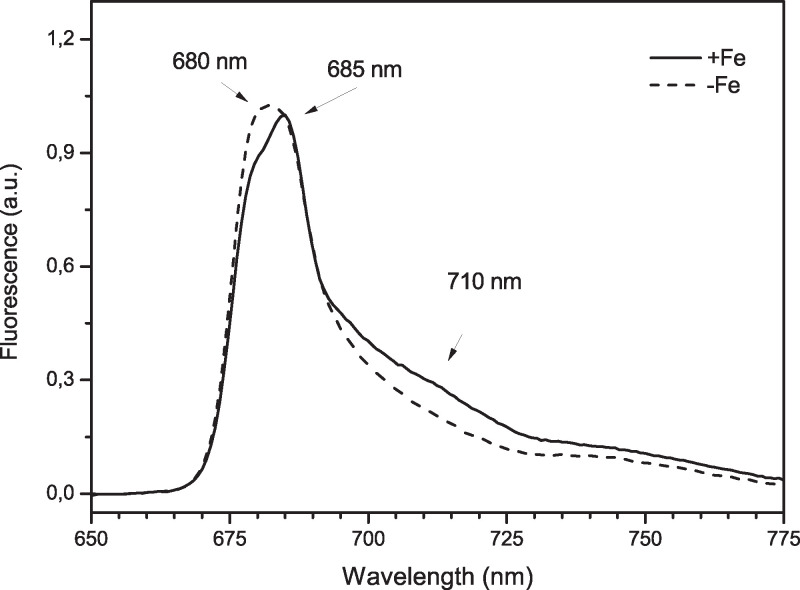
Fluorescence emission spectra obtained at 77 K for *B. natans* grown in the presence of iron (+Fe) and in the absence of iron (−Fe). Fluorescence was induced by excitation of chlorophyll *a* at 455 nm. The spectra presented here were normalized to the PSII fluorescence emission maxima (685 nm). Data represent typical curves.

Because of its high iron content, photosystem I (PSI) is known to be a prime target of iron deficiency. By means of 77 K fluorescence emission spectroscopy, we observed a clear drop in the PSI/PSII ratio (fluorescence at 710 nm [*F*_710_]/*F*_685_) under iron-limited conditions (Fig. 7). Furthermore, 77 K data revealed a significant increase of fluorescence at 680 nm (*F*_680_) (Fig. 7) suggesting reduced energy transfer to the reaction centers due to partial decoupling of light-harvesting antennae (34) or, alternatively, due to enhanced antenna abundance under iron-limited conditions. Following iron replenishment, the *F*_710_/*F*_685_ ratio as well as *F*_680_ maximum regenerated very slowly, as the first noticeable changes in the 77 K spectra were visible only after 24 h after iron enrichment (data not shown).

**FIG 8:**
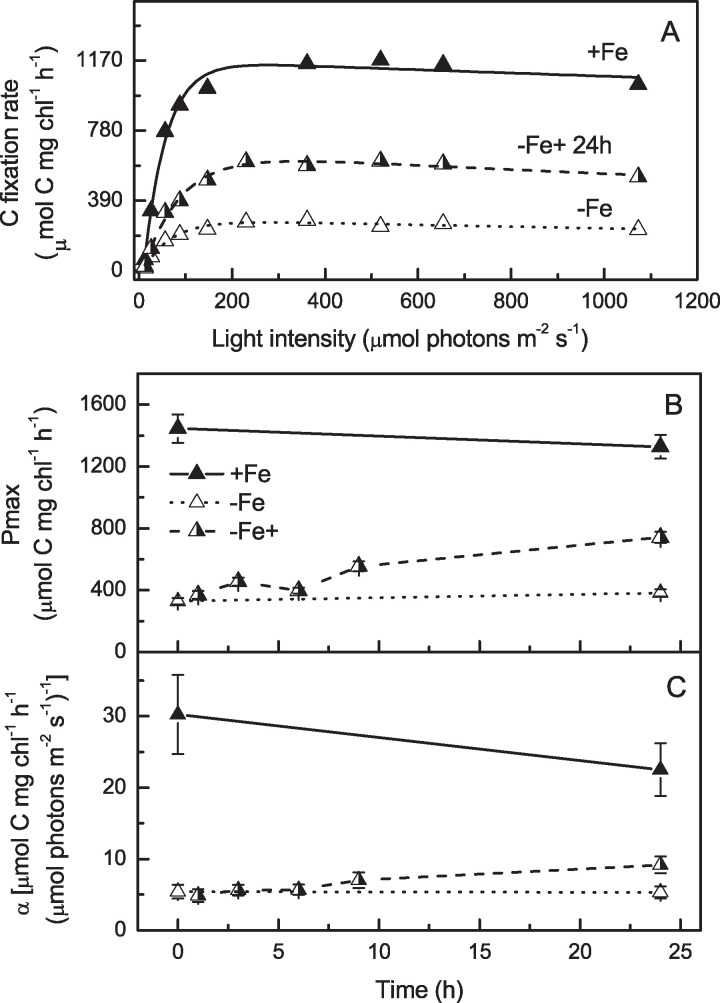
Carbon fixation rates of *B. natans* grown in the presence of iron (+Fe), in the absence of iron (−Fe), and after iron supply of iron limited cells (−Fe+). (A) Carbon fixation rate as a function of irradiance. Measured data are represented by symbols; data fitting is represented by lines. For better overview only the curve 24 h after iron supply is shown. (B and C) Maximum rate of C fixation (*P*_max_) (B) and photosynthetic efficiency (α) (C) measured during the 24 h following iron supplementation. Values are means and SD derived by data fitting (see Materials and Methods).

To test how the effects of the iron deficit on *B. natans* PSI physiology are reflected in downstream processes, we recorded photosynthetic light response (PI) curves based on radiolabeled carbon-14 incorporation (Fig. 8). The pigment-normalized maximum rate of C fixation (*P*_max_) was strongly affected by iron supply. *P*_max_ was 4.4-fold higher in iron-sufficient cells than iron-starved ones (Fig. 8B). The recovery of *P*_max_ was slow compared to the response of PSII parameters. The first signs of regeneration were visible only after 9 h of iron repletion, and after 24 h, *P*_max_ reached merely half of the rate of C fixation in control cultures grown under iron-rich conditions. Photosynthetic efficiency (α), measured from the initial slope of a PI curve, was affected by lack of iron even more and responded to iron enrichment even more slowly (Fig. 8C).

## DISCUSSION

The ability to respond to changing growth conditions is particularly important for organisms occupying a nutritionally poor environment. Our study has shown that *B. natans* cells possess powerful mechanisms that respond to the sudden switch from iron limitation to iron abundance. Among the most highly responding proteins were two phytotransferrins, whose levels decreased as early as 3 h after iron enrichment. For comparison, rapid transcriptional responsiveness to iron enrichment was also observed in the model diatom Thalassiosira pseudonana, with completion of gene expression downregulation within 3 h after iron resupply (35). The fast decrease in protein levels observed in our study suggests either quick protein turnover or an effective posttranslational regulation in *B. natans*. Such a rapid and strong decline of phytotransferrin levels and the corresponding reduction of iron uptake upon iron enrichment raises a question as to how iron is stored within the cell, considering the fact that *B. natans* lacks the iron storage protein ferritin. Whether iron is stored in an organelle, as was suggested for the diatom *Thalassiosira* (36), remains to be elucidated. Candidate homologues of NRAMP (natural resistance-associated macrophage protein; hypothesized to be responsible for transporting iron out of a vacuole in *Thalassiosira* species [36]) or VIT1/CCC1, involved in iron transport to the vacuole, are present in the *B. natans* genome (23).

Intriguingly, *CREG1* exhibited the strongest response to iron limitation alongside phytotransferrins. While CREG1 has been linked to iron limitation in *P. tricornutum*, where this gene is located within one of the identified iron-regulated clusters (5) and colocalizes with phytotransferrin to the vicinity of the chloroplast (37), it was surprising to see it behave similarly in the distantly related chlorarachniophyte. A duplicated CREG1-like domain was identified in an iron-responsive protein of Thalassiosira oceanica, an oceanic diatom well adapted to iron limitation (38). To assess the possible universal role of CREG1 in iron stress responses among all phytoplankton, we looked for gene and transcript abundances in environmental data sets. Here, for the first time, we provide evidence that CREG1 is universally linked with iron limitation in the ocean, since its mRNA levels are significantly upregulated in metazoans as well as photosynthetic eukaryotes from all branches of the tree of life under conditions of iron starvation (Fig. 4). The exact function of this protein is not known, but studies in human cell lines, mice, and *Drosophila* show that it is involved in proliferation, differentiation, and cell senescence (31). We propose that CREG1 may be responsible for the inhibition of *B. natans* growth during iron limitation. It was recently suggested that in *P. tricornutum*, CREG1 functions as a ferric reductase involved in the iron uptake machinery (37). However, experimental evidence for this is insufficient, and a direct link to iron metabolism has yet to be demonstrated in any species. Its ubiquity in iron homeostasis across different phytoplankton species calls for further experiments to investigate the mechanistic role of this protein.

Flavodoxins and ferredoxins are well-known markers of iron stress in photosynthetic organisms. The adaptive value of flavodoxins under iron limitation lies in their functional replacement of the iron-containing ferredoxins as electron carriers in the photosynthetic electron transport chain (13). Consistent with this, the level of the two *B. natans* ferredoxins was higher in iron-rich cells while the expression of flavodoxin was lower, and the same patterns appeared at the transcript level when we analyzed abundance in *Tara* Oceans environmental samples. Interestingly, while the expression of flavodoxin dropped relatively quickly after iron supply to iron-limited cells, with significant change observed after 9 h, the upregulation of ferredoxins was much slower, and in the case of FDX2, the abundance of the protein 24 h after iron addition to *B. natans* cells in culture was more similar to that found in iron-limited cells than to that in cells grown in long-term iron-replete conditions. This is in strong contrast to the effect of iron enrichment on the abundance of phytotransferrins and the CREG1 protein. Similarly, the iron-containing cytochrome *c*_6_, responsible for electron transport between cytochrome *b*_6_*f* and PSI, displayed a slow response to iron, being upregulated only after 24 h. These differences in the response to iron enrichment between different proteins, such as between these electron transporters and proteins involved in iron uptake, are difficult to reconcile. We believe that this phenomenon most probably arises from multiple factors affecting the regulation of protein levels, e.g., the differences between transcriptional and posttranslational regulation, variations in protein turnover rates, and diversity in the sensing of stimuli such as the simple intracellular iron concentration compared to a complex redox state of the photosynthetic apparatus.

In total, 77 proteins displayed a >1.5-fold change in abundance 24 h after iron resupply to iron-limited *B. natans* cells, including several upregulated iron-dependent proteins or components of iron-rich complexes. While we could not assign accurate functions in iron metabolism for all of them, there were hints to suggest a relation to iron metabolism in some cases. The iron-induced increase in the expression of an acyl carrier protein (Table 1) is in agreement with the observed iron-regulated changes in transcript levels of one of the plastidial acyl carrier proteins in Arabidopsis thaliana, which had led the authors of a previous study to suggest a function of this protein as a hub between light, nitrogen, and iron deficiency (39). Moreover, an acyl carrier protein was shown to be directly involved in the mitochondrial iron-sulfur assembly machinery and probably plays a role in the modulation of this pathway (40). Perhaps a more direct link to iron can be argued for the CobW domain-containing protein, whose expression decreased nearly 10-fold 24 h after iron supplementation and was not detected in cells grown under long-term iron-rich conditions. This protein is a member of the COG0523 family, whose functions are very diverse, ranging from the incorporation of cobalt into the tetrapyrrole ring during cobalamin biosynthesis in prokaryotes to activation of Fe-type nitrile hydratase, all functions thus being related to metal homeostasis (29). Considering the high abundance of the members of this family in organisms throughout the tree of life (being present in most sequenced genomes) (29), this homologue from *B. natans* as well as homologues from other phytoplankton species deserve attention in the field of trace element research.

Since there is considerable interest in the function of the nucleomorph of *B. natans*, we considered whether any of the nucleomorph-encoded proteins were regulated by iron. We detected two, albeit only slightly affected by iron status, which corroborates findings from previous transcriptome studies in *B. natans*. For example, genome-wide diurnal expression profiling (20) showed that the expression of more than 7,000 genes significantly oscillated along diurnal/cell cycles, while only two of them were encoded by the nucleomorph. The authors of that study thus hypothesized that nucleomorph genes are not involved in controlling diurnal cycles (20). Similarly, only slight changes in nucleomorph gene expression were observed in response to light stress, suggesting the predominant role of the nucleus in transcriptional control (21). Our results therefore support the observation that regulation of nucleomorph genes appears to be uncoupled from the general homeostasis of the *B. natans* cell.

As expected, photosynthesis of low-iron-adapted *B. natans* cells was affected mostly at the level of PSI, which is the most iron-rich component of the light reactions of photosynthesis (each complex requires 12 iron atoms to function). Fluorescence emission spectra obtained at 77 K indicated a relative decrease of the PSI/PSII ratio in response to iron depletion. Downregulation (Table 1) of ferredoxins (PetF-1 and PetF-2) along with the PSI subunit PsaE, involved in NADP^+^ reduction via interaction with ferredoxin-NADP^+^ reductase (FNR), resulted in substantially reduced rates of photosynthetic carbon fixation. The recovery of carbon fixation capacity was slow, with half times estimated to be more than 35 h. Contrary to PSI, at the level of PSII, the rate of photosynthetic electron transport was not affected by iron concentrations. Gross oxygen evolution rates per cell were independent of iron and in all samples were around 0.023 ± 0.002 pmol O_2_ cell^−1^ h^−1^, which is typical for *B. natans* cultivated under iron-replete conditions and similar light conditions (21).

While iron deficiency did not cause cell chlorosis, iron availability affected the arrangement of the light-harvesting antennae in *B. natans*. The appearance of a *F*_680_ peak in the 77 K fluorescence emission spectrum (Fig. 7) indicated that part of the light-harvesting complex (LHC) is functionally disconnected and/or that antenna abundance in Fe-deficient cells is significantly enhanced. Disconnection of the LHCI antenna from PSI is known to occur under low-iron conditions in the chlorophyte model alga Chlamydomonas reinhardtii (41, 42), where it was proposed to be regulated via the PSI-K subunit of PSI, the accumulation of which is influenced by the activity of iron-requiring aerobic oxidative cyclase (42). But while uncoupled LHCI in C. reinhardtii peaks at 705 nm, the increased 680-nm emission maximum in *B. natans* observed here (Fig. 5) points rather to a loosely coupled LHCII (reviewed in reference 34). High-repetition-rate fluorometry measurements further revealed significant enlargement of the functionally coupled PSII antenna under iron deficiency (Fig. 6C). The effective PSII antenna cross-section (σ_PSII_) increased by ∼1.9 nm^2^ per PSII in iron-limited cells. It should be noted that σ is the product of optical cross-section (i.e., physical antenna size itself) and the efficiency of photochemistry in PSII (usually expressed as *F_v_*/*F_m_*). Given that *F_v_*/*F_m_* during iron deficiency was low (0.35) compared to that under iron-replete conditions (0.5), we can estimate that the physical antenna size of PSII under iron deficiency had more than doubled. A similar phenomenon was reported for the distantly related model diatom *P. tricornutum* (43) and the green alga C. reinhardtii, for which it was suggested that increased antenna size serves as a buffer/storage of pigment to allow for fast recovery after iron resupply (41). In summary, reduced PSI/PSII ratio resulted in enhanced antenna abundance at PSII in iron-deficient cells, which increased σ and light absorption capacity. The excitation energy flow from the increased antenna to the PSII core was less efficient, and the antenna seemed to be partly functionally disconnected from PSII, which resulted in increased *F*_680_ fluorescence emission at 77 K.

With the increased capacity for light absorption by PSII but inhibited the PSII acceptor side by substantially downregulated PSI, *B. natans* engaged photoprotective NPQ to safely dissipate excess absorbed energy to heat. An increase of photoprotective NPQ corresponded with the upregulation in expression of the light stress-related LHXC proteins, as shown by the proteomic data (LI818 2 and LI818 1 proteins in Table 1). Aside from dissipation of excitation energy to heat, excess energy after charge separation in PSII may have been dissipated by electron transfer to O_2_ via alternative terminal oxidases (44). Electron transfer to O_2_ via plastid terminal oxidase (PTOX) has been reported as a common strategy in oligotrophic environments to keep PSII oxidized and thus minimize photodamage when levels of the iron-rich cytochrome *b*_6_*f* complex and PSI are reduced (45). This situation is well confirmed by our proteomic data. By maintaining the PSII antennae and preventing excessive photodamage to PSII through LI818-induced NPQ and diversion of electrons to O_2_, the PSII photochemical quantum yield could be quickly restored after resupply of iron, with a half time of full recovery around 24 h.

Compared to iron-rich PSI, which recovered rather slowly after iron enrichment, the PSII response to iron resupply was more rapid, with a half time around 20 h. The processes protecting the photosynthetic electron transport chain from photodamage, namely, NPQ but also depressed *F_v_*/*F_m_*, reacted promptly even though the functional antenna size remained enlarged for several more hours. Collectively, the combination of our proteomic analysis with physiological experiments demonstrates that *B. natans* is well adapted to dynamically respond to a changing iron environment.

Taken together, our results provide a comprehensive description of the response of *B. natans* to iron enrichment. Combining detailed proteomic and functional analyses, we showed that the main strategies employed to use iron efficiently under widely contrasting conditions of availability involve strict regulation of the iron uptake machinery, replacement of iron-containing proteins by functional homologues, and dynamic changes in photosynthetic electron flow to maintain functional PSII even under reduced levels of the most iron-rich components of the electron transport chain. We further reveal the likely importance of CREG1 and COG0523 in iron homeostasis, not only in *B. natans* but also in other phytoplankton.

## MATERIALS AND METHODS

### Cell culture.

*B. natans* (CCMP2755) (kindly provided by John M. Archibald, Dalhousie University) was grown at 18°C under a 12-h/12-h light (50 μmol m^−2^ s^−1^)/dark regimen in a modified f/2 medium, as described previously (27). The composition of the growth medium was as follows: 40 g/liter sea salts (Sigma), 2.66 mg/liter NH_4_NO_3_, 75 mg/liter NaNO_3_, 22.8 mg/liter Na_2_SiO_3_ · 5H_2_O, 15 mg/liter NaH_2_PO_4_, 1 ml of vitamin stock (20 mg/liter thiamine HCl, 1 mg/ml biotin, 1 mg/ml B_12_), and 1 ml of trace metal stock (200 mg/liter MnCl_2_ · 4H_2_O, 40 mg/liter ZnSO_4_ · 7H_2_O, 20 mg/liter Na_2_MoO_4_ · 2H_2_O, 14 mg/liter CoCl_2_ · 6H_2_O, 10 mg/liter Na_3_VO_4_ · nH_2_O, 10 mg/liter NiCl_2_, 10 mg/ml H_2_SeO_3_). The medium was buffered with 1 g/liter HEPES (pH 7.5).

Time course physiological experiments were performed under constant light. Iron-rich conditions were achieved by the addition of 100 nM ferric citrate (1: 20).

### Comparative proteomic analysis.

Label-free whole-cell proteomic analysis was performed in independent biological triplicates for each condition. Sample preparation, liquid chromatography coupled with mass spectrometry, and data analysis and quantification were performed using the method described in reference 46. Proteomic data analysis details are summarized in Table S1.

10.1128/mSystems.00738-20.2 TABLE S1 Description of proteomic data analysis. Download Table S1, DOC file, 0.1 MB. Copyright © 2021 Kotabova et al. 2021 Kotabova et al. This content is distributed under the terms of the Creative Commons Attribution 4.0 International license.

### Iron acquisition.

The incorporation of iron into protein complexes was analyzed by blue native PAGE as described in reference 28. At each time point specified in Fig. 2, cells grown under different iron conditions were washed with 5 mM EDTA to remove extracellular iron, transferred to fresh cultivation medium, and incubated for 1 h with 1 μM ^55^Fe citrate (1:20). Iron uptake was stopped by the addition of 5 mM EDTA. The cells were then washed three times with ice-cold cultivation medium and disrupted by sonication in the presence of 1% digitonin, and protein complexes were separated by blue native PAGE using the Novex native PAGE 4 to 16% bis-Tris gel system (Invitrogen) according to the manufacturer’s protocol. The gels were vacuum dried and autoradiographed for 7 days using a BAS-IP TR 2025 E tritium storage phosphor screen (GE Healthcare Life Sciences) and visualized by Typhoon FLA 7000 (GE Healthcare Life Sciences).

### Analysis of biogeographical and environmental distribution of *B. natans* in *Tara* Oceans data.

*Tara* Oceans performed a worldwide sampling of plankton between 2009 and 2013, which generated data sets with different approaches, including rRNA gene-based metabarcoding (24, 25) and metagenomics/metatranscriptomics (26). In order to analyze the biogeography of *B. natans*, we retrieved the operational taxonomic units (OTUs) assigned to this species from the 18S rRNA gene (V9 region) metabarcoding data set from samples obtained from surface waters across 144 geographical sites (24, 25) (https://zenodo.org/record/3768510#.Xraby6gzY2w). The *B. natans* barcode abundance in each sample was normalized to the barcode abundance of eukaryotic phytoplankton. Graphs were plotted with R library ggplot2 (47).

We also compared the *B. natans* metabarcoding abundance with the environmental data collected during *Tara* Oceans expeditions. Measurements of temperature were carried out at each station with a vertical profile sampling system (CTD rosette) and Niskin bottles following the sampling package described in reference 48. Chlorophyll *a* concentrations were measured using high-performance liquid chromatography (49, 50). Phosphate concentrations were determined using segmented flow analysis (51). Nitrate and nitrite concentrations were measured using a Satlantic ISUS nitrate sensor (48). Iron levels were derived from a global circulation model (52).

### Identification of *Bigelowiella* flavodoxin and ferredoxin genes in the *Tara* Oceans eukaryote unigenes catalogue.

We carried out a search for sequences coding for the 2Fe-2S iron-sulfur cluster binding domain (PF00111) and for flavodoxin (PF00258) in the *Tara* Oceans eukaryote gene atlas (MATOU-v1) (26). We used HHMer v3.2.1 with the gathering threshold option (http://hmmer.org/), retrieving a total of 62 and 17 sequences, respectively, assigned to Chlorarachniophytes. For a better taxonomic assignment and for removal of the nonphotosynthetic homologues, we performed a phylogenetic placement of the translated sequences on the reference ferredoxin and flavodoxin phylogenetic trees described in reference 53. First, they were aligned against the corresponding reference alignment using the option –add of MAFFT version 6 with the G-INS-I strategy (54). The output alignment was trimmed in both N- and C-terminal regions to maintain the reference alignment limits. The resulting alignment was used for building the phylogeny with PhyML version 3.0 (55). Four categories of rate variation were used. The starting tree was a BIONJ tree, and the type of tree improvement was subtree pruning and regrafting. Branch support was calculated using the approximate likelihood ratio test (aLRT) with a Shimodaira-Hasegawa-like (SH-like) procedure. The sequences were classified according to their grouping in monophyletic branches with statistical support of >0.7 with reference sequences of the same functional or taxonomic group. The final number of *Bigelowiella*-like sequences corresponding to photosynthetic ferredoxin was 29, and that for photosynthetic flavodoxin was 9. We retrieved the metatranscriptomic read abundance for these sequences and compared them to iron seawater concentrations extracted from the ocean biogeochemical model PISCES2 (56).

### Identification of CREG1 in the *Tara* Oceans eukaryote unigenes catalogue.

We carried out a search for sequences containing the Pfam domain pyridoxamine 5′-phosphate oxidase (PF13883), present in CREG1, using HMMer v3.2.1 as previously described, in the sequenced genomes available at Integrated Microbial Genome (IMG) (http://img.jgi.doe.gov) (57) and in the sequenced transcriptomes from the Marine Microbial Eukaryote Transcriptome Sequencing project (MMETSP) (58). Our search retrieved 882 genes from bacteria and eukaryotes (0 from archaea and viruses). These sequences were used for building a protein similarity network for the PF13883 domain using the EFI-EST tool (59) (score cutoff = 22) and Cytoscape visualization (60), which allowed us to identify two major subfamilies within the proteins containing the pyridoxamine 5′-phosphate oxidase domain. One subfamily contains sequences from all the major photosynthetic eukaryotes (including *B. natans*, Emiliania huxleyi, Fragilariopsis cylindrus, *Aureococcus* sp., *Monosigma* sp., and *P. tricornutum*, where CREG1 was annotated in their genomes) as well as a few eukaryotic heterotrophs. The second subfamily contains proteins from both bacteria and eukaryotes and, alongside the PF13883 domain, contained an extra domain called DUF2470.

We repeated the HMMer search for PF13883 in MATOU-v1 (26), retrieving a total of 6,291 sequences. We used the protein similarity network to parse between these sequences, keeping 4,855 sequences aggregated into the CREG1 subfamily (which lacked the DUF2470 domain).

We retrieved the metagenomic and metatranscriptomic read abundance for these sequences and for 21 conserved single-copy ribosomal proteins from eukaryotes (used for normalization) (26) and compared their ratio to iron seawater concentrations extracted from the ocean biogeochemical model PISCES2 (56).

As plankton were separated into discrete size fractions using a serial filtration system (61), the *Tara* Oceans data sets used here are structured into four main size fractions, from 0.8 to 5 μm (including small phytoplankton such as *B. natans*), followed by 5 to 20 μm, 20 to 180 μm, and 180 to 2,000 μm (which is particularly enriched in metazoans but also includes phytoplankton such as large diatom chains) (61).

### Identification of a new subfamily of COG0523 metallochaperones in the *Tara* Oceans eukaryote unigenes catalogue.

We carried out a HMMer search for sequences containing the Pfam domain CobW (PF02492), present in the COG0523 family, in the reference genome and transcriptome databases IMG and MMETSP as described above. We obtained a total of 25,369 sequences from bacteria, archaea, and eukaryotes (0 from viruses). We also retrieved the sequences used for defining the 15 subfamilies of COG0523 by Haas et al. (29). The CobW domain of all these sequences was used for building a protein similarity network as described above (score cutoff = 65), which recovered the iron-responsible protein from our proteomic experiments (jgi|Bigna1|43984|e_gw1.87.5.1) as part of the same cluster as subfamilies 1 and 2. Therefore, we built a phylogeny for the Pfam domain region of all sequences of this cluster as well as the sequences from subfamilies 3 to 15 used by Haas et al. (29). We first reduced redundancy with CD-HIT version 4.6.4 using an 80% identity cutoff (62) and then aligned the sequences with MAFFT version 6 using the G-INS-I strategy (54). The output alignment was trimmed in both the N- and C-terminal regions to maintain the reference alignment limits. The phylogenetic tree was generated with PhyML version 3.0 (55) as previously described.

We repeated the HMMer search for PF02492 in MATOU-v1 (26), retrieving a total of 22,591 sequences. We used the above protein similarity network and the phylogeny to keep only the sequences grouping with protein 43984 and to improve their taxonomic assignation, resulting in only 39 sequences of this new subfamily. We retrieved the metagenomic and metatranscriptomic read abundances for these sequences and for 21 conserved single-copy ribosomal proteins from eukaryotes (used for normalization) (26) and compared their ratio with the predicted iron seawater concentrations, as described in the section above.

### Physiological experiments.

Specific growth rates (μ; per day) were determined from cell abundance measured using a calibrated Coulter Counter (Multisizer 4; Beckman, Indianapolis, IN, USA) equipped with a 50-μm aperture, and calculated as (ln *c* − ln *c*_0_)/(*t* − *t*_0_), where *c* is the cell concentration and *t* is time measured in days.

Chlorophylls were quantified as described by Jeffrey and Humphrey (63). The aliquots of algal suspension were collected on GF/F filters (Whatman, England), soaked in 90% acetone, and stored at −20°C for 24 h. The absorption spectra of the extracts were then measured using a UV/visible-light (Vis) spectrophotometer (Unicam UV 550; Thermo Spectronic, United Kingdom).

Photosynthetic oxygen evolution was measured by using a Hansatech DW1 oxygen electrode chamber (Hansatech Instruments Ltd., Narborough, United Kingdom), coupled to a PSI OxyCorder 401 A/D signal transducer equipped with PSI OxyWin software (Photon Systems Instruments, Brno, Czech Republic). Oxygen evolution was measured at 18°C in the presence of 1 mM sodium bicarbonate under saturating light irradiance (700 μmol photons m^−2^ s^−1^) provided by LEDs of the Act2 system (Chelsea Technologies Group Ltd., Surrey, United Kingdom). The values presented here were calculated from the slope of O_2_ evolution at a given irradiance plus the slope of respiratory O_2_ utilization measured in the dark.

Fluorescence parameters were measured after 10 min dark adaptation. The maximum quantum yield of photochemistry (*F_v_*/*F_m_*) was measured using AquaPen AP 100 (Photon Systems Instruments, Brno, Czech Republic) using a multiple turnover blue (λ = 450 nm) saturating flash. The nonphotochemical fluorescence quenching (NPQ) and effective PSII antenna cross-section (σ_PSII_) were determined by high-repetition-rate fluorometry with a FastOcean fluorometer (Chelsea Technologies Group Ltd., Surrey, United Kingdom) using a single-turnover induction protocol of 100 blue (450 nm) flashlets over ca. 200 μs (2 μs flashlet pitch). This was done for the 10 levels of actinic light intensities (0 to 595 μmol photons m^−2^ s^−1^). NPQ was calculated as (*F_m_* − *F_m_*′)/*F_m_*′ (Stern–Volmer formalism), where *F_m_* and *F_m_*′ are the maximum fluorescence measured in the dark and light (200 μmol m^−2^ s^−1^), respectively. To derive σ_PSII_, data were fitted by using FastPRO software (Chelsea Technologies Group, Surrey, United Kingdom).

Low-temperature fluorescence emission spectra were measured using an SM 9000 spectrophotometer (Photon Systems Instruments, Brno, Czech Republic) at an excitation wavelength of 455 nm. Aliquots of a 50-μl culture sample were placed in a copper sample holder and cooled to 77 K in liquid nitrogen in a Dewar with a transparent finger for low-temperature fluorescence emission measurements. A blank spectrum was measured using plain culture medium, and this measurement was later subtracted from each of the sample measurements. The fluorescence spectra were normalized to the 685-nm peak, which represented the photosystem II emission maximum.

Photosynthetic carbon fixation was determined by short-term incubations with radioactively labeled sodium bicarbonate (MP Biochemicals, USA; final concentration of 1 μCi ml^−1^) according to Lewis and Smith (64). The samples were incubated with the ^14^C isotope in the temperature-controlled (18°C) photosynthetron for 30 min at light intensities ranging from 13 to 1,100 μmol photons m^2^ s^−1^. Triplicate samples for background counts (with buffered formalin) and total counts (with ethanolamine) were prepared at the start. After incubation, samples were immediately acidified with 200 μl 17.5% HCl (vol/vol) and left to degas on an orbital shaker to purge unincorporated label. After 24 h degassing, Eco-Lite scintillation cocktail (MP Biomedicals, CA, USA) was added to each sample to determine its radioactive decay using a Tri-Carb 2810 TR liquid scintillation analyzer (PerkinElmer, MA, USA). Dissolved inorganic carbon concentrations were determined in a cell-free medium by the Gran titration technique described by Butler (65). Chlorophyll-specific carbon fixation rates were plotted against irradiance, and photosynthesis-irradiance curves were then fitted using *f* = *y*_0_ + *P*_max_ × {1 − exp[−(α × *x*)/*P*_max_]} × exp(β × *x*/*P*_max_) to derive the maximum chlorophyll-specific carbon fixation rate (*P*_max_) and photosynthetic efficiency (α) according to the model of Eilers and Peeters (66).
