# Supplementary material for: Complex Response of the Chlorarachniophyte Bigelowiella natans to Iron Availability
Source: mSystems. 2021 Feb 9;6(1):e00738-20. doi: 10.1128/mSystems.00738-20 (PMC7883536; doi:10.1128/mSystems.00738-20)
Supplement: TABLE S1 [file mSystems.00738-20-st001.doc]

**Table S1**

| **Sample preparation** | Cells were lysed in 100mM TEAB containing 2% SDC (95°C for 5 min); 20 µg of protein was used for MS sample preparation (determined by BCA protein assay kit (Thermo)); cysteines were reduced by 5mM tris(2-carboxyethyl)phosphine (60°C for 60 min) and blocked with 10mM methyl methanethiosulfonate (23°C 10 min); overnight digestion by trypsin (trypsin/protein ratio 1/30) was carried out at 37°C and followed by acidification with 1% TFA (final concentration). SDC was removed by extraction to ethylacetate and peptides were desalted (1, 2) |
| --- | --- |
| **Enzyme specificity** | Trypsin/P with max 2 miscleavages and minimal and maximal peptide length 7 and 25 amino acids respectively |
| **Trap column** | C18 PepMap100, 5 μm particle size, 300 μm x 5 mm (Thermo Scientific) 4 min, 18 μl/min; loading buffer (2% acetonitrile, 0.1% trifluoroacetic acid, water); elution with mobile phase gradient from 4% to 35% in 120 min; 1 ug of sample was loaded on the column |
| **LC-MS column** | Nano Reversed phase column (EASY-Spray column, 50 cm x 75 µm ID, PepMap C18, 2 µm particles, 100 Å pore size) |
| **Mobile phases** | Mobile phase buffer A (water and 0.1% formic acid); mobile phase B (acetonitrile and 0.1% formic acid) |
| **Peptides analysis** | Gas-phase ions were analyzed by a Thermo Orbitrap Fusion (Q-OT- qIT, Thermo Scientific); survey scans from 350 to 1400 m/z in orbitrap; resolution - 120K (200 m/z); target ion count - 5 × 10^5^; tandem MS - 1,5 Th quadrupol; HCD fragmentation collision energy - 30; rapid scan MS analysis in the ion trap; MS2 ion count target - 10^4^; max injection time - 35 ms; precursors with charge state 2–6 were sampled for MS2; dynamic exclusion duration - 45 s with a 10 ppm tolerance; top speed mode; 2 s cycles |
| **Peak list, search engine** | All data were processed by MaxQuant 1.6.1.0 with built-in search engine Andromeda, which was also used for peak list generation, peptides assignment to proteins and data normalization (according to (3)); MS1 maximum peak intensity was used for quantification |
| **Sequence database** | Bigelowiella natans chloroplasts uniprot database containing 575 entries (15.2.2019) and nucleomorph uniprot database containing 287 entries (15.2.2019) (4) and Bigelowiella natans filtered proteins database from JGI genome portal containing 21 708 entries (19.5.2010) |
| **Data analysis** | Proteins were automatically annotated using the eggNOG database (http://eggnogdb.embl.de/) and manually curated using HHpred (https://toolkit.tuebingen.mpg.de/); Analysis was performed using Perseus 1.6.1.3 software (5); statistical relevance was estimated using students t-test with Benjamini-Hochberg’s correction (FDR = 0.05) |
| **Fixed modifications** | Carbamidomethyl on cysteine (+57.02146) |
| **Variable modifications** | Oxidation (M), Acetyl (Protein N-term) |
| **Mass tolerance for precursor ions** | 20 ppm for first search (before recalibration) and 4.5 ppm for main search (after recalibration) |
| **Mass tolerance for fragment ions** | 0.5 Da |
| **False Discovery Rates at Peptide and Protein levels** | 1 % at both peptide and protein level |

1. Masuda, T., Tomita, M., and Ishihama, Y. (2008). Phase transfer surfactant-aided trypsin digestion for membrane proteome analysis. J. Proteome Res. *7*, 731–740.

2. Rappsilber, J., Mann, M., and Ishihama, Y. (2007). Protocol for micro-purification, enrichment, pre-fractionation and storage of peptides for proteomics using StageTips. Nat. Protoc. *2*, 1896–1906.

3. Cox, J., Hein, M.Y., Luber, C.A., Paron, I., Nagaraj, N., and Mann, M. (2014). Accurate Proteome-wide Label-free Quantification by Delayed Normalization and Maximal Peptide Ratio Extraction, Termed MaxLFQ. Mol. Cell. Proteomics *13*, 2513–2526.

4. Gilson, P.R., Su, V., Slamovits, C.H., Reith, M.E., Keeling, P.J., and McFadden, G.I. (2006). Complete nucleotide sequence of the chlorarachniophyte nucleomorph: Nature’s smallest nucleus. Proc. Natl. Acad. Sci. U. S. A. *103*, 9566–9571.

5. Tyanova, S., Temu, T., Sinitcyn, P., Carlson, A., Hein, M.Y., Geiger, T., Mann, M., and Cox, J. (2016). The Perseus computational platform for comprehensive analysis of (prote)omics data. Nat. Methods *13*, 731–740.
